# Meta-Structure Hull Design with Periodic Layered Phononic Crystals Theory for Wide-Band Low-Frequency Sound Insolation

**DOI:** 10.3390/ma16124429

**Published:** 2023-06-16

**Authors:** Fuxi Zhang, Xinyi Sun, Wei Tao, Shiming Wang, George T. Flowers, Qingsong Hu, Oleg Gaidai

**Affiliations:** 1 College of Engineering Science and Technology, Shanghai Ocean University, Shanghai 201306, China; 2 Department of Mechanical Engineering, Auburn University, Auburn, AL 36849, USA

**Keywords:** acoustic metamaterial, low-frequency isolation, selective frequency tunneling, Periodic Strato-Shaped Phononic Crystals (PSPC)

## Abstract

The hulls of marine vehicles are generally very effective at attenuating airborne acoustic noise generated by their powertrains. However, conventional hull designs are generally not very effective at attenuating wide-band low-frequency noise. Meta-structure concepts offer an opportunity for the design of laminated hull structures tailored to address this concern. This research proposes a novel meta-structure laminar hull concept using periodic layered Phononic crystals to optimize the sound insolation performance on the air–solid side of the hull structure. The acoustic transmission performance is evaluated using the transfer matrix, the acoustic transmittance, and the tunneling frequencies. The theoretical and numerical models for a proposed thin solid-air sandwiched meta-structure hull indicate ultra-low transmission within a 50-to-800 Hz frequency band and with two predicted sharp tunneling peaks. The corresponding 3D-printed sample experimentally validates the tunneling peaks at 189 Hz and 538 Hz, with 0.38 and 0.56 transmission magnitudes, respectively, with the frequency band between those values showing wide-band mitigation. The simplicity of this meta-structure design provides a convenient way to achieve acoustic band filtering of low frequencies for marine engineering equipment and, accordingly, an effective technique for low-frequency acoustic mitigation.

## 1. Introduction

In recent years, there has been a considerable research interest in sound-attenuating metamaterials regarding the noise of ship and underwater vehicles. In terms of theoretical research, the plate and cylindrical housing are usually used as simplified models of acoustic radiation characteristics. Junger studied the acoustic radiation characteristics of cylindrical housings immersed in a variety of acoustic media with ring-shaped stiffeners [1]. Bleich and Baron defined vacuum modes on the basis of Junger, arranging a certain number of longitudinal bones, ring ribs, or stiffeners of ship and underwater vehicle structures, which was a common method of providing structural strength and stiffness, further complicating the analysis of structural acoustic characteristics of ships and underwater vehicles [2]. Maidanik conducted a study on the acoustic vibration characteristics of infinite plate structures with periodic arrangement of linear supports and concluded that the acoustic radiation efficiency of the structure increased significantly below a specific frequency point [3]. Mace proposed a sonic-resonance characterization analysis of the supporting plate structure of the periodic distribution line in parallel with each other [4]. Yoshikawa et al. made a theoretical study on the acoustic response of a bilayer cylindrical shell with a fluid medium between shells [5]. Li and Yan propose hybrid expanded elastic metamaterials that may be a candidate for subwavelength-scale vibration-attenuating structures [6]. At present, metamaterials have been extensively studied in marine ships and show great potential in engineering applications such as underwater gliders [7], mechanical drivetrains [8], and ships [9].

In terms of the study of radiated noise underwater, Pilon et al. established the acoustic–vibration coupling relationship on the surface of cylindrical structures according to the relationship between sound pressure and vibration velocity of large curvature structures and improved the computational efficiency of structural vibration acoustic radiation by using Kirchhoff approximation conditions [10,11]. Zhu et al. reviewed the basic characteristics and development history of sound absorption, sound insulation, and decoupling. Using underwater acoustic equipment to explore the unknown marine environment is one of the important means to understand and utilize the ocean [12]. Since underwater acoustic equipment is often used in various marine environments, it will inevitably produce some subtle irregular vibrations, and the authors believe that the application of underwater acoustic metamaterials is key to improving the engineering performance indicators, mainly including underwater acoustic detection and acoustic stealth [13]. Heaney, K.D. et al. applied isometric elements to the Helmholtz equation discrete and gave a formula for calculating acoustic radiation on the surface of non-smooth structures which was of great significance for improving the efficiency of solving radiated noise in surface structures [14]. A method to achieve them by inserting transverse membranes with sub-wavelength periods along the channel was proposed by Benchabane et al. that demonstrated a new type of acoustic tunneling [15]. A one-dimensional limited-size phonon crystal (PC) lattice composed of specially configured single cells was proposed to achieve broadband high-performance filtering in a relative high frequency range [16]. A finite phononic lattice filter composed of impedance mirror elements was proposed that shifted the frequency range of previous work [17]. In general, Bragg scattering [18] and local resonance [19] are the main mechanisms for generating bandgaps, and the bandgap of local resonance mechanisms is usually lower than that of Bragg scattering mechanisms when the lattice constants are quantitative and equal [20]. Metamaterials refer to artificial periodic microstructures with singular characteristics, and the unusual physical properties are mainly derived from the microstructure of geometry, rather than its material composition, and the band structure and transport response of metamaterials are analyzed [21]. The emergence of acoustic metamaterials has provided some new techniques, proposing the concept of designing a phononic lattice with a bandgap in the audio range, using the wave attenuation band caused by Bragg scattering to minimize harmful vibration and noise [22]. Porous materials are lightweight and come in a variety of shapes and perform well in regard to absorbing high-frequency noise. In order to improve the sound-absorption effect of porous materials on medium- and low-frequency noise, it is proposed to use filled structures, gradient porous materials, and porous metal materials [23,24,25]. Mohammad et al. studied the sound-absorption properties of coir [26]. In the above research, the sound-absorption performance of porous materials was improved, and although porous metal materials have stable mechanical properties, they will produce high production costs. Therefore, porous materials are difficult to use alone in most environments and can be used as a good auxiliary materials, and the research methods mainly include connecting the resonator cavity in series with the porous material and filling the cavity in the resonant cavity with the porous material [27,28,29,30,31]. Over the past decades, acoustical metamaterials have been extensively studied. Researchers have realized that traditional materials cannot achieve certain acoustic functionalities. Dong et al. reviewed the progress in underwater acoustic metamaterials, including underwater acoustic stealth, using metamaterials to reduce the scattering cross-section of objects and achieve acoustic stealth; underwater beam formation, using metamaterial structures to control sound propagation direction, enabling acoustic beam scanning and splitting; underwater meta-surfaces and phase engineering, using subwavelength structures on material surfaces to manipulate the phase and amplitude of sound, achieving functions such as focusing and holograms; underwater topological acoustics, using topological metamaterials with bandgaps to control sound propagation; and underwater acoustic metamaterial absorption, using metamaterials to enhance sound absorption over a wider frequency range compared to traditional materials [32]. Defects and boundaries in these metamaterials enable robust sound transmission channels and edge states. 

Based on the transmission matrix method, Chen and Cao, through the transfer matrix method, calculated the transmission coefficient of the layered structure composed of glass and water [33]. Phonon propagation at the interface of the periodic super-lattice layer was studied perpendicular to the finite period. Seiji Mizuno and Shin-ichiro Tamura theoretically studied the resonance transmission of phonons in a bi-barrier system [34,35]. M. Červenka and M. Bednařík verified the reconstruction of the reflection or transmission of sound waves induced by the axial temperature distribution of the fluid and proposed the corresponding numerical algorithm [36]. A new impedance change scheme, the special configuration of aluminum-based metamaterials with in-homogeneity double-seam hole, and the actual wave transmission performance of metamaterials were studied numerically and experimentally [37]. Ilinskii et al. established a one-dimensional model in acoustic resonators and revealed the dependence of resonance hardening and softening on the geometry of the resonator [38]. The shape of the acoustic resonator was optimized to maximize the amplitude of the sound pressure. Červenka and Bednařík achieved a vocal bandpass filter by inserting a shape waveguide element between the two parts of the sound transmission line [39,40]. Chen et al. proposed a structural optimization algorithm to successfully achieve UAT with high rectification efficiency to reduce wave propagation in metamaterials [41]. Zhang et al. developed a system topology optimization method based on the Material Field Series Expansion (MFSE) framework for the design of omnidirectional bandgap acoustic metamaterials to broaden the omnidirectional bandgap [42]. Li and Yan studied the bandgap characteristics and longitudinal elastic wave attenuation and proposed a triangular structure to generate a wide bandgap based on the theoretical lumped mass and finite element method in the mass model [6]. Almeida et al. using shape optimization techniques to expand the bandgap while maintaining unit cell length on the order of tens of millimeters [22]. Periodic resonators such as Helmholtz resonators and membrane cavity resonators improve sound transmission losses in pipes by creating a wide bandgap [43,44,45].

The previous research studies have conducted a large number of experimental, theoretical and FEA simulation studies on the sound transmission characteristics at relatively high frequencies, rather than the acoustic attenuation of layered periodic phononic lattices at ultra-low and wide band frequencies. Meanwhile, tunneling peaks featured stopband that can trace back the characteristics of the original noise have paid less attention. In order to improve the ultra-low wide band stop of marine vehicle noises, as well as to characterize the tunneling frequencies of the attenuator, this research work proposed a deliberate Periodic Strato-Shaped Phononic Crystals (PSPC)-implemented meta-structure hull design for practical marine vehicle applications that is lower in weight, easy in fabrication, and without sophisticated substructures. The analytical derivations, corresponding simulation results, and experimental validations are detailed in the following sections.

## 2. Theoretical Derivations

### 2.1. Transfer Metrics of Acoustic–Phononic Lattices

**Figure 1:**
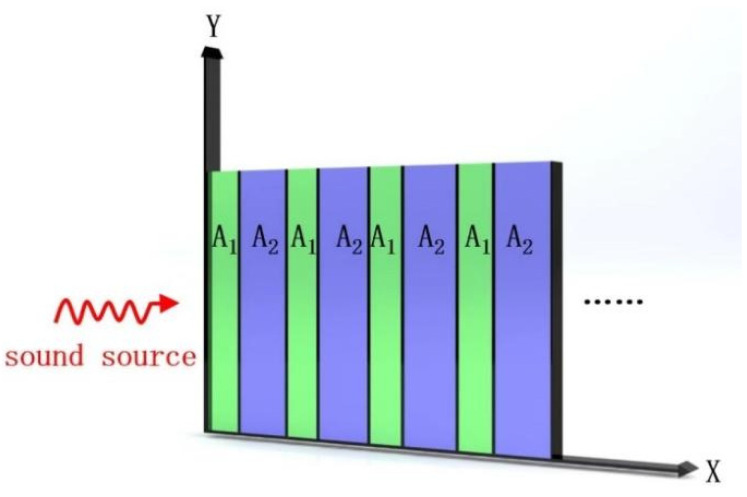
Schematic of an ideal Strato-Shaped Phononic Crystals (PSPCs).

One-dimensional acoustic–phononic lattices consisting of repeating dual-material units can be modeled using the form of the wave equation for elastic wave propagation. A single lattice unit is constructed of laminated $A_{1}$ and $A_{2}$ materials. The acoustic transmission characteristics of the lattices, treated as a continuous mass media, can be derived from correlations of particle velocity and acoustic pressure. Figure 1 provides a conceptual illustration of the phonon lattice units alternating continuously in the x-direction, with thicknesses $d_{A_{1}}$ and $d_{A_{2}}$. The vibration-induced acoustic wave propagates along the x-axis and satisfies the equilibrium condition shown in Equation (1) [46]:

(1) $$\rho \left(x\right)\frac{\partial^{2}u\left(x,y,t\right)}{\partial t^{2}}=\mu \left(x\right)\frac{\partial^{2}u\left(x,y,t\right)}{\partial x^{2}}$$

where, in a period distance of $d=d_{A_{1}}+d_{A_{2}}$, $u\left(x,y,t\right)$ denotes the amplitude of particle motion in the x direction; ρ(x) is the corresponding material density; and $\mu \left(x\right)$ is the Lamé coefficient of the selected materials. The Lamé coefficient, $\mu \left(x\right)$, is crucial to understanding and predicting wave motion and equilibrium in medium. It characterizes a material’s fundamental elastic properties, determines wave speeds, appears in the basic equations of motion, and links microscale and macroscale behavior [47].

The time harmonic mode solution of Equation (1) is of the following form:

(2) $$u\left(x,t\right)=u\left(x\right)exp\left[-i\omega t\right]$$

The wave equation can then be derived by substituting Equation (2) into Equation (1):

(3) $$\mu \left(x\right)\frac{\partial^{2}u\left(x\right)}{\partial x^{2}}-\rho \left(x\right)\omega^{2}u\left(x\right)=0$$

For isotropic material layers, the wave propagation in the material can be treated as consisting of a transmitted portion and a reflected portion, resulting in $u\left(x\right)=Ae^{ikx}+Be^{-ikx}$. Here, k is the wave number, v is the phase speed of sound across materials, and $\mu =\rho v^{2}$ is the Lamé coefficient. The particle-amplitude-correlated stress is $s\left(x\right)=\mu \left(x\right)\frac{\partial u\left(x\right)}{\partial x}$, and the resulting transmission and reflection can be obtained form the solution of Equation (4):

(4) $$\left\{\begin{array}{c} u_{i}\left(x\right)=c_{i}^{t}e^{ik_{i}x}+c_{i}^{r}e^{-ik_{i}x}\\ s_{i}\left(x\right)=i\omega {Z_{i}(c}_{i}^{t}e^{ik_{i}x}-c_{i}^{r}e^{-ik_{i}x}) \end{array}\right.$$

where i is the lattice layer index, $c_{i}^{t}$ and $c_{i}^{r}$ are the amplitudes of the transmitted and reflected portions of the wave, $k_{i}$ is the wave number for each specific layer, $Z_{i}=\rho_{i}v_{i}$ is the acoustic impedance, $v_{i}$ is the phase speed of sound, and $\omega =k_{i}v_{i}$ is the angular frequency.

At the interface of two different mass densities, the continuity condition can be expressed as follows:

(5) $$W_{i}\left(x\right)=\left(\begin{array}{c} u_{i}\left(x\right)\\ s_{i}\left(x\right) \end{array}\right)=h_{i}\left(x\right)C_{i}=\left[\begin{array}{cc} e^{ik_{i}x} & e^{-ik_{i}x}\\ i\omega {Z_{i}e}^{ik_{i}x} & -i\omega {Z_{i}e}^{-ik_{i}x} \end{array}\right]\left[\begin{array}{c} c_{i}^{t}\\ c_{i}^{r} \end{array}\right]$$

Moreover, the transfer matrix of layer $A_{1}$ can be obtained from $W_{i}\left(x\right)$, with $\alpha_{1}=k_{A_{1}}d_{A_{1}}$. The resulting transfer matrix is $\left[\begin{array}{cc} \cos\alpha_{1} & (\sin\alpha_{1})/\left(\omega Z_{A_{1}}\right)\\ -\omega Z_{A_{1}}\sin\alpha_{1} & \cos\alpha_{1} \end{array}\right]$. Likewise, the transfer matrix of $A_{2}$ can be obtained in a similar fashion. Consequently, the transfer matrix, $T_{A}$, of a single period lattice unit is as follows:

(6) $$T_{A}=\left[\begin{array}{cc} \lambda_{A} & \sigma_{A}/\left(\omega Z_{A_{1}}\right)\\ \omega Z_{A_{1}}\xi_{A} & \mu_{A} \end{array}\right]$$

where we have the following:

(7) $$\left\{\begin{array}{c} \begin{array}{c} \lambda_{A}=\cos\alpha_{1}\cdot \cos\alpha_{2}-{[Z}_{A_{1}}(\sin\alpha_{1}\cdot \sin\alpha_{2})]/Z_{A_{2}}\\ \sigma_{A}=\sin\alpha_{1}\cdot \cos\alpha_{2}+{[Z}_{A_{1}}(\cos\alpha_{1}\cdot \sin\alpha_{2})]/Z_{A_{2}} \end{array}\\ \begin{array}{c} \xi_{A}=-\sin\alpha_{1}\cdot \cos\alpha_{2}-[Z_{A_{2}}(\cos\alpha_{1}\cdot \sin\alpha_{2})]/Z_{A_{1}}\\ \mu_{A}=\cos\alpha_{1}\cdot \cos\alpha_{2}-\left[Z_{A_{2}}\left(\sin\alpha_{1}\cdot \sin\alpha_{2}\right)\right]/Z_{A_{1}} \end{array} \end{array}\right.$$

where $\alpha_{1}$, $\alpha_{2}$, $Z_{A_{1}}$ and $Z_{A_{2}}$ are the relevant to material properties and physical thicknesses. The corresponding derivations are detailed in the Appendix. For *N* period phononic lattices, the transfer matrix in series is ${T_{A}\left(N\right)=(T_{A})}^{N}$, where we have the following:

(8) $$T_{A}\left(N\right)=\left[\begin{array}{cc} {[(\lambda }_{A}{-\mu }_{A})S_{A}\left(N\right)]/2+C_{A}\left(N\right) & {(\sigma }_{A}S_{A}\left(N\right))/\left(\omega Z_{A_{1}}\right)\\ \omega Z_{A_{1}}\xi_{A}S_{A}\left(N\right) & -{[(\lambda }_{A}{-\mu }_{A})S_{A}\left(N\right)]/2+C_{A}\left(N\right) \end{array}\right]$$

**Figure 2:**
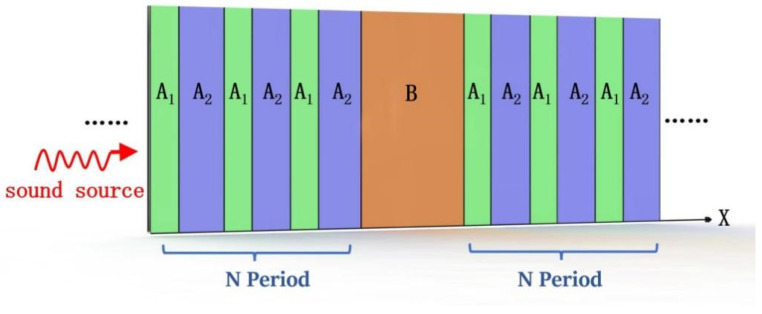
Schematic of PSPC meta-structure. The green regions represent solid panels, the blue regions represent slit areas, and the orange region represents air cushion B.

The specific values of $S_{A}\left(N\right)$ and $C_{A}\left(N\right)$ depend on the phase speed and the density at a given position. Using this modeling approach, it is also possible to include the effects of other materials, such as an air cushion, indicated by layer B, as shown in Figure 2, for the laminated *N* period. The transfer matrix for layer B with a thickness of $T_{B}$ is the same as the transfer matrix for a single material structure, where ${Th}_{z}$ is the thickness of layer B, denoted by $T_{B}$:

(9) $$T_{B}=\left[\begin{array}{cc} \cos\gamma & \left(\sin\gamma \right)/\left(\omega Z_{A_{1}}\right)\\ -\omega Z_{A_{1}}\sin\gamma & \cos\gamma \end{array}\right]$$

and where $\gamma =k_{B}{Th}_{z}=k_{A_{1}}{Th}_{z}$. The transfer matrix for the combined sandwiched structure is obtained as shown in Equation (10):

(10) $${T=T}_{A}\left(N\right)T_{B}T_{A}\left(N\right)$$

where

(11) $$\begin{array}{l} T_{A}\left(N\right)={\left(T_{A}\right)}^{N}\\ =\left[\begin{array}{cc} {[(\lambda }_{A}{-\mu }_{A})S_{A}\left(N\right)]/2+C_{A}\left(N\right) & {(\sigma }_{A}S_{A}\left(N\right))/\left(\omega Z_{A_{1}}\right)\\ \omega Z_{A_{1}}\xi_{A}S_{A}\left(N\right) & -{[(\lambda }_{A}{-\mu }_{A})S_{A}\left(N\right)]/2+C_{A}\left(N\right) \end{array}\right] \end{array}$$

where $T_{11}$, $T_{12}$, $T_{21}$, and $T_{22}$ represent the following (10):

(12) $$T=\left[\begin{array}{cc} T_{11} & T_{12}\\ T_{21} & T_{22} \end{array}\right]$$

Assuming that the motion amplitude of the downstream phonons also satisfies the continuity condition at the various interfaces,$W_{i}\left(x\right)$, of Equation (5), the transmission rate ($T_{r}$) can be expressed as follows:

(13) $$T_{r}=4/\left[{\left(T_{2}-T_{3}\right)}^{2}+{\left(T_{1}+T_{4}\right)}^{2}\right]$$

where $T_{1}=T_{11}$, $T_{2}={wZ_{A_{1}}T}_{12}$, $T_{3}=T_{21}/\left(wZ_{A_{1}}\right)$, and $T_{4}=T_{22}$.

### 2.2. Transfer Matrix Model of Phononic Lattice Meta-Structure

The material parameters of the two media are brought into the transfer matrix of the specific model. The transmission coefficient can be derived from $T_{11}$, $T_{12}$, $T_{21}$, and $T_{22}$, as exhibited in Equation (12), which corresponds to the wholesome meta-structure properties of the PSPC. In this research work, the corresponding parameters are $\rho_{A_{1}}$ = 1.205 kg/m^3^, $v_{A_{1}}$ = 343 m/s, $\rho_{A_{2}}$ = 1150 kg/m^3^, $v_{A_{2}}$ = 2650 m/s, ${Th}_{z}$ = 0.02 m, $d_{A_{1}}$ = 0.002 m, and $d_{A_{2}}$ = 0.005 m. The transition matrix of a periodic phononic lattice ($T_{A}$) varies with $\alpha_{i}=k_{i}d_{i}\;(i=1,2)$ of the layer $A_{1}$ and $layerA_{2}$. To obtain the consequent 3 periodic phononic crystal transfer matrices $T_{A}\left(N=3\right)$, insert the parameters of the B field into Equation (8) to obtain $T_{B}$. Therefore, the transfer matrix for the entire model can be determined by ${T=T}_{A}\left(N\right)T_{B}T_{A}\left(N\right)$. The determination of $S_{A}\left(N\right)$ and $C_{A}\left(N\right)$ in Equation (8) depends on the size of the parameter $\left|\left(\lambda_{A}{+\mu }_{A}\right)/2\right|$; the $\lambda_{A}$ and $\mu_{A}$ values are expressed in Equation (7). Substituting $\alpha_{1}$, $\alpha_{2}$, $Z_{A_{1}}$, and $Z_{A_{2}}$ into $\left|\left(\lambda_{A}{+\mu }_{A}\right)/2\right|$ for the threshold calculation, the result is that $\left|\left(\lambda_{A}{+\mu }_{A}\right)/2\right|<1$, and it claims that the value of $S_{A}\left(N\right)$ and $C_{A}\left(N\right)$ should follow the expressions $S_{A}\left(N\right)=\sin\left(N\theta_{A}\right)/\sin\theta_{A}$ and $C_{A}\left(N\right)=cos(N\theta_{A})$ (here assigning $\cos\left(\theta_{A}\right)=\left(\lambda_{A}{+\mu }_{A}\right)/2$) [35]. Correspondingly, $T_{11}$, $T_{12}$, $T_{21}$, and $T_{22}$ can be determined by Equation (14):

(14) $$\left\{\begin{array}{c} \begin{array}{c} T_{11}=\left(\cos\gamma D-\sigma_{A}S_{A}\left(N\right)\sin\gamma \right)\cdot D+{wZ}_{A_{1}}\xi_{A}S_{A}\left(N\right)[\left(\sin\gamma \cdot D\right)/\left({wZ}_{A_{1}}\right)+\left(\sigma_{A}S_{A}\left(N\right)\cos\gamma \right)/\left({wZ}_{A_{1}}\right)]\\ T_{12}=\left[\left(\sin\gamma \cdot D+\sigma_{A}S_{A}\left(N\right)\cos\gamma \right)\cdot F+\sigma_{A}S_{A}\left(N\right)\left(\cos\gamma \cdot D-\sigma_{A}S_{A}\left(N\right)\sin\gamma \right)\right]/\left(wZ_{A_{1}}\right) \end{array}\\ \begin{array}{c} T_{21}={wZ}_{A_{1}}[\xi_{A}S_{A}\left(N\right)\left(\cos\gamma \cdot F+{\xi_{A}S}_{A}\left(N\right)\sin\gamma \right)-\left(\sin\gamma \cdot F-\xi_{A}S_{A}\left(N\right)\cos\gamma \right)\cdot D]\\ T_{22}=\left(\cos\gamma \cdot F+{\xi_{A}S}_{A}\left(N\right)\sin\gamma \right)\cdot F-\left[(\sigma_{A}S_{A}\left(N\right))({wZ}_{A_{1}}\sin\gamma \cdot F-{wZ}_{A_{1}}\xi_{A}S_{A}\left(N\right)\cos\gamma )\right]/\left({wZ}_{A_{1}}\right) \end{array} \end{array}\right.$$

where

(15) $$\left\{\begin{array}{c} {D=C}_{A}\left(N\right)+E\\ E=\left[\left(\lambda_{A}{-\mu }_{A}\right)\cdot S_{A}\left(N\right)\right]/2\\ F=C_{A}\left(N\right)-E \end{array}\right.$$

Taking the manipulation $T_{1}=T_{11}$, $T_{2}={wZ_{A_{1}}\cdot T}_{12}$, $T_{3}=T_{21}/\left(wZ_{A_{1}}\right)$, and $T_{4}=T_{22}$, Equation (16) can be obtained for deriving the sound transmission rate ($T_{r}$) of the entire PSPC model.

(16) $$\left\{\begin{array}{c} \begin{array}{c} T_{1}=\left(\cos\gamma D-\sigma_{A}S_{A}\left(N\right)\sin\gamma \right)\cdot D+\xi_{A}S_{A}\left(N\right)[(\sin\gamma \cdot D)+(\sigma_{A}S_{A}\left(N\right)\cos\gamma )]\\ T_{2}=\left(\sin\gamma \cdot D+\sigma_{A}S_{A}\left(N\right)\cos\gamma \right)\cdot F+\sigma_{A}S_{A}\left(N\right)\left(\cos\gamma \cdot D-\sigma_{A}S_{A}\left(N\right)\sin\gamma \right) \end{array}\\ \begin{array}{c} T_{3}=\xi_{A}S_{A}\left(N\right)\left(\cos\gamma \cdot F+{\xi_{A}S}_{A}\left(N\right)\sin\gamma \right)-\left(\sin\gamma \cdot F-\xi_{A}S_{A}\left(N\right)\cos\gamma \right)\cdot D\\ T_{4}=\left(\cos\gamma \cdot F+{\xi_{A}S}_{A}\left(N\right)\sin\gamma \right)\cdot F-[\sigma_{A}S_{A}\left(N\right)(\sin\gamma \cdot F-\xi_{A}S_{A}\left(N\right)\cos\gamma )] \end{array} \end{array}\right.$$

### 2.3. Finite-Element Analysis Setup

**Figure 3:**
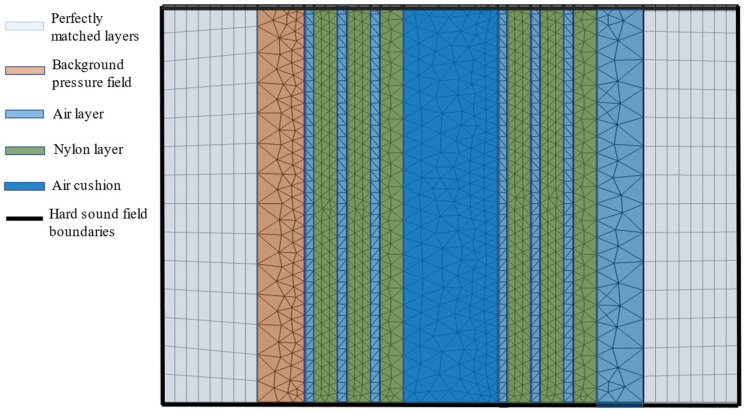
FEA model of PSPC meta-structure. The width of the air cushion and the period number, *N*, can be adjusted as needed.

As shown in Figure 2 and Figure 3, layer $A_{1}$ is air, while layer $A_{2}$ is rigid nylon. The combination of layers $A_{1}$ and $A_{2}$ form phononic crystals with a period of 1. The model demonstrated in Figure 3 consists of an air-cushion region in the center, with periodic structures of period *N* = 3 on either side. PMLs are applied at the outer ends of the model to absorb all outgoing waves and eliminate reflections. This allows the central sample region, highlighted in green and blue, to be studied without interference from reflected waves. The PMLs create an open-boundary condition by simulating a non-reflective open space outside the model region. The most typical use of PMLs is to model open space in the studied region by essentially simulating non-reflective boundaries. The PML domain functions in the constant coefficient wave equation, where the field describes the energy of radiation. The PML acts as a near-ideal non-reflective or open boundary. Both the scaling factor and the proportional curvature parameters of the PMLs are 1.

The FEA model emitted plane waves from the background pressure field (in red) toward the PSPC, at a pressure amplitude of 2 Pa. The mesh size was set to be smaller than or equal to $c/f_{max}/5$ based on the speed of sound (c) and the maximum frequency ($f_{max}$), with a minimum cell size of 0.0054 cm. The PML had 8 units to fully absorb outgoing waves. With these settings, the simulation model could accurately study the interaction of the emitted plane waves with the PSPC.

## 3. Theoretical Results

### 3.1. Periodic Phononic Lattices without an Air Layer

Recalling the derivations from the last section, we can see that the frequency-related acoustic characteristics are highly correlated with the structure of the PSPC element and the periods of PSPC. The transfer matrix, without considering the cushion layer, meets the prediction of Equation (11). Moreover, where $T_{1}=D=C_{A}\left(N\right)+\left[\left(\lambda_{A}{-\mu }_{A}\right)\cdot S_{A}\left(N\right)\right]/2$, $T_{2}=\sigma_{A}S_{A}\left(N\right)$, $T_{3}=\xi_{A}S_{A}\left(N\right)$, and $T_{4}=F=C_{A}\left(N\right)-\left[\left(\lambda_{A}{-\mu }_{A}\right){\cdot S}_{A}\left(N\right)\right]/2$. The transmission rate is expressed in terms of ${T_{r}}^{’}$:

(17) $${T_{r}}^{’}=4/\left[{\left(\sigma_{A}S_{A}\left(N\right)-\xi_{A}S_{A}\left(N\right)\right)}^{2}+4{C_{A}\left(N\right)}^{2}\right]$$

**Figure 4:**
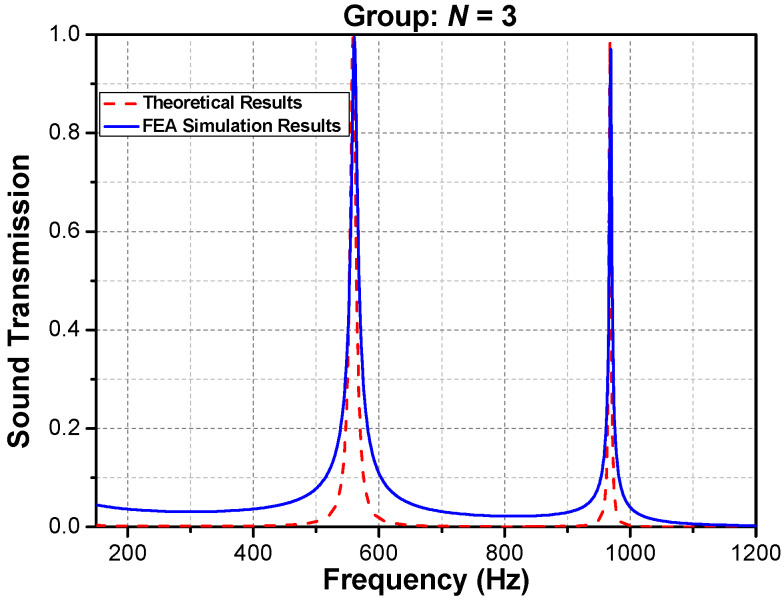
Theoretical and FEA simulation results at *N* = 3.

With a periodic number N=3 for the phononic crystal, the preliminary verification in Figure 4 shows that the finite element analysis (FEA) simulation results closely match the theoretical predictions, with high transmission peaks at approximately 549 Hz and 968 Hz. The remaining portions exhibit an approximate square–concave shape, with transmission rates dipping below 0.03. It is possible to predict high transmission tunneling peaks passing through the wide soundproofing band of the phononic crystal, the principles of which are explained in detail as follows.

In order to further verify of the theoretical model, the FEA-simulated acoustic transmission rate for the periods of N=2,3,4,5,6,and 7 is discussed, and it is found that the number of transmission peaks increases along with the increasing PSPC cycle. In a specific frequency band, the tunneling frequency point interval decreases against the increase of the number of cycles.

**Figure 5:**
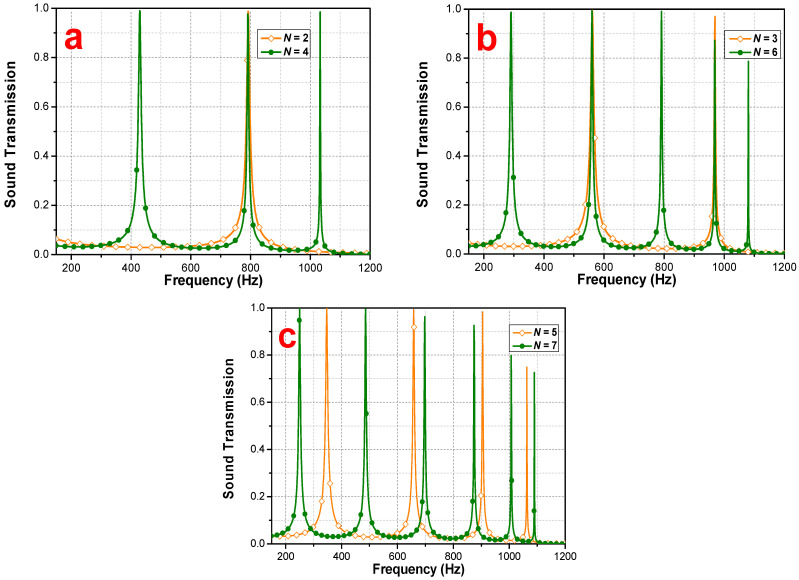
FEA-simulated results of sound transmission at period *N* = 2, 3, 4, 5, 6, and 7. (**a**) *N* = 2 vs. *N* = 4; (**b**) *N* = 3 vs. *N* = 6; (**c**) *N* = 5 vs. *N* = 7.

By observing Figure 5, we can see that FEA-simulated transmissions of N=2 and N=4 have the same peaks at about 800 Hz, and those of N=3 and N=6 have the same peaks at about 600 Hz and 1000 Hz. Comparing N=2 and N=4 with N=3 and N=6, it is found that doubling the period will lead to a new tunneling frequency between two original tunnelings. For example, N=4 has a new tunneling at 430 Hz regarding N=2, and N=6 leads to new tunneling peaks around 300 Hz and 800 Hz regarding N=3. The occurrence of this phenomenon is correlated to the sub-matrix, $T_{n}$, of the transmission rate, $T_{r}^{\prime }$.

**Figure 6:**
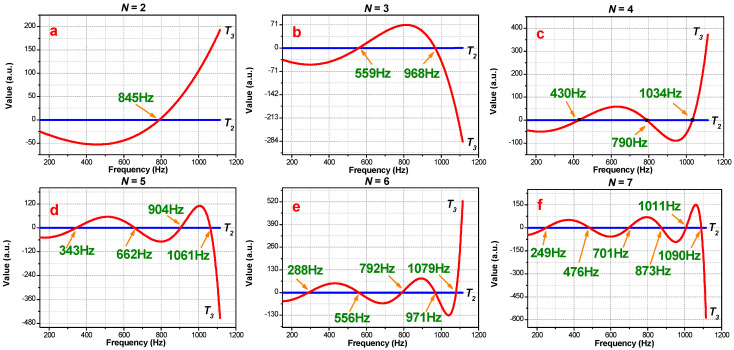
Theoretical predictions of tunneling frequencies calculated using the determinant of sub-matrix $T_{2}\;\mathrm{and}\;T_{3}$ of Equation (16): (**a**) period *N* = 2, (**b**) period *N* = 3, (**c**) period *N* = 4, (**d**) period *N* = 5, (**e**) period *N* = 6, and (**f**) period *N* = 7.

In Figure 6, this particular correlation of sub-matrices and period variations of PSPC-induced tunneling frequencies was studied. The intersection node numbers of $T_{2}$ and $T_{3}$ increase along with the PSPC period number, N, rising up. The $T_{3}$ curves all follow the manner of oscillating before the last intersection node and of non-convergence in the rest portion. Comparing the theoretical and FEA simulation results, we see that the intersection nodes of $T_{2}$ and $T_{3}$ agree well with the tunneling frequencies where the sound can transmit through the whole PSPC meta-structure. The intersection frequency of $T_{2}$ and $T_{3}$ at N=2 meets the tunneling frequency at about 845 Hz. For N=3, $T_{2}$ and $T_{3}$ intersect at 2 nodes that meet the tunneling frequency at approximately 559 Hz and 968 Hz; for N=4, $T_{2}$ and $T_{3}$ intersect at 2 positions that agree with the tunneling frequencies at 430 Hz, 790 Hz, and 1034 Hz, respectively.

Regardless of the peak at 0 Hz, the relationship between the number of tunneling peaks ($N_{a}$) and the number of PSPC periods (N) is $N_{a}=N-1$, which is also determined by the transmittance expression denominator in Equation (17). The second term of the denominator is two orders less than the first term, so the transmittance mainly relies on the first term; that is, when the first term comes to $\sigma_{A}S_{A}\left(N\right)-\xi_{A}S_{A}\left(N\right)=0$, the sound wave is supposed to transmit through PSPC with little attenuation, and the transmittance is close to 1. Therefore, it can be determined that the tunneling frequencies are firmly consistent with the intersection frequencies between $T_{2}$ and $T_{3}$.

**Figure 7:**
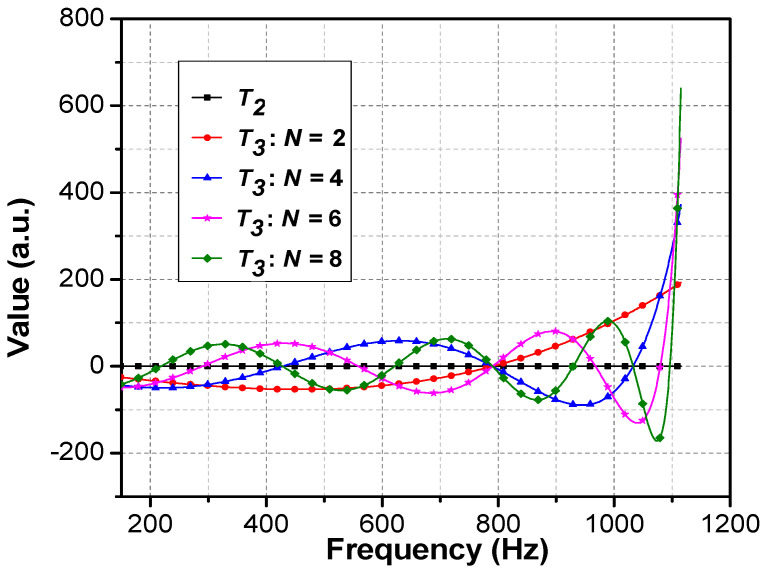
*T*_2_ and *T*_3_ intersections at periods of *N* = 2, 4, 6, and 8.

Figure 7 illustrates the theoretical results of varying N versus intersection nodes that predicted each doubled N will lead to a new node, as well as to the tunneling frequency, inserting in between the original adjacent nodes, and this property is constantly true for even numbers of period N.

The volatility of the $T_{3}$ curve and the uniqueness of the intersection nodes ensure that the tunneling peak can accurately characterize the stopbands of the PSPC, which can rapidly trace back the period (N) at certain properties of layers $A_{1}$ and $A_{2}$.

### 3.2. Periodic Phononic Lattices with an Air Cushion

**Figure 8:**
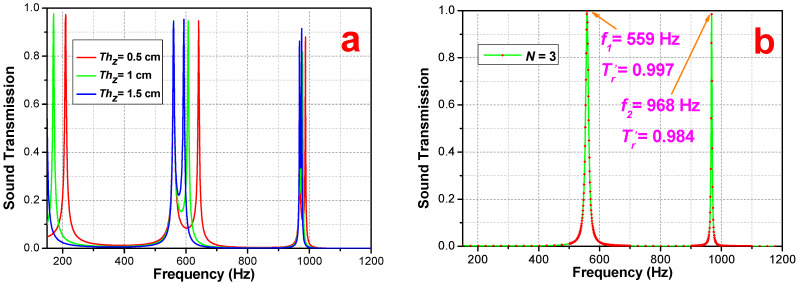
Sound transmission with air-cushion thickness: (**a**) ${Th}_{z}$ = 0, 1.5, 1, and 2 cm; (**b**) theoretical results at *N* = 3 without ${Th}_{z}$.

For the case that PSPC has an air cushion B in the middle, referring to Figure 2, the air cushion is sandwiched by two groups of three-layered (N=3) sub-PSPCs, where the total period (N) is still 6. Figure 8 shows the tunneling frequencies and stopbands when the air-cushion thickness, ${Th}_{z}$, is specified from 0 cm to 1.5 cm, with a 0.5 cm increment. It is clear that the air-cushion thickness, ${Th}_{z}$, at a specific N controls the shift of tunneling frequency. Reviewing the tunneling clusters around 590 Hz and 980 Hz, we see that the tunneling peak of ${Th}_{z}$ = 1.5 cm is located in the center, with other peaks being locating symmetrically around it.

**Figure 9:**
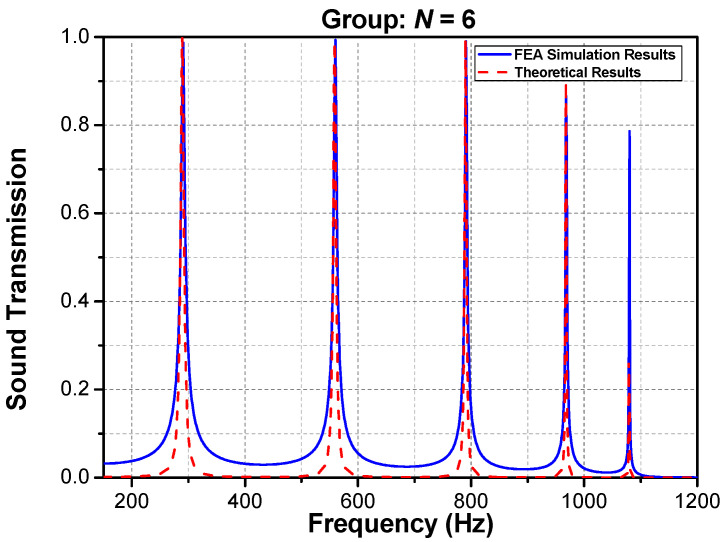
Theoretical- and FEA-simulation results of sound transmission at *N* = 6 without ${Th}_{z}$.

Figure 9 illustrates the tunneling-frequency deviation varying with the change in thickness. Along with ${Th}_{z}$ from 0 to 2 cm, the tunneling frequencies converge to a specific frequency. This convergence frequency exactly meets the corresponding tunneling frequency of one group sub-PSPC, or equally to a single three-layered PSPC. The theoretical results refer to the ideal situation; that is, if the transmission at the tunneling frequency point is close to 1, the stopbands stay close to 0. The corresponding FEA simulation results meet the theoretical predicted frequencies, but with reasonable deviations in amplitude.

**Figure 10:**
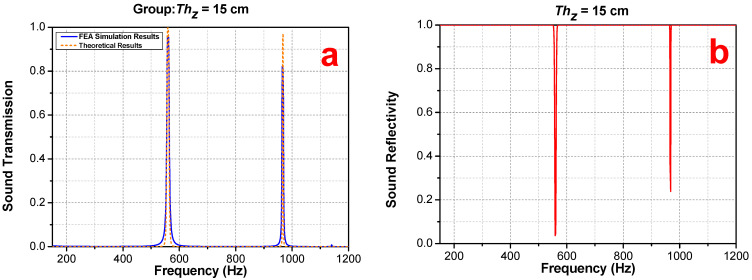
(**a**) Results of sound transmission at ${Th}_{z}$ = 15 cm, *N* = 6; (**b**) results of sound reflectivity at ${Th}_{z}$ = 15 cm, *N* = 6.

**Figure 11:**
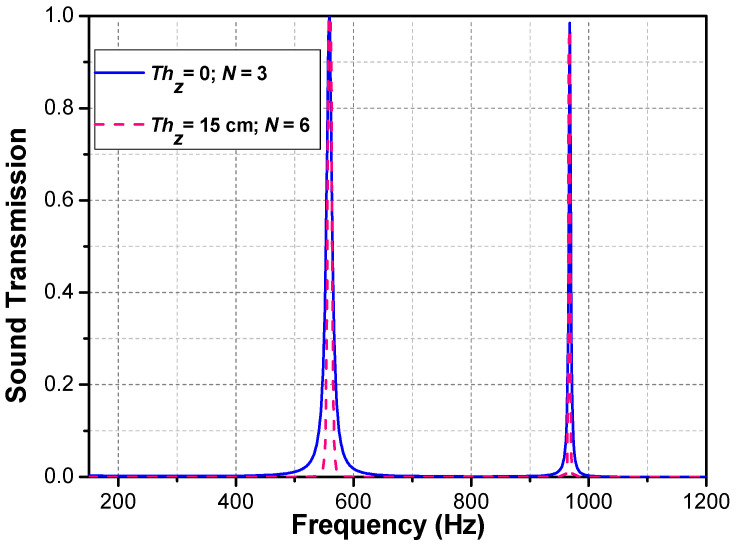
The blue curve represents the acoustic transmission of N=3 without the air cushion; the red curve represents the acoustic transmission with the air-cushion thickness of ${Th}_{z}$ = 15 cm and period N=6.

Figure 10 demonstrates the FEA validation regarding the theoretical prediction of symmetrical PSPC in the case of ${Th}_{z}$ = 15 cm and N=6. It can be found that the acoustic reflectivity is far below 0.1 at the first tunneling peak frequency shown in Figure 10, and the reflectivity at the second tunneling frequency is close to 0.2, but at other frequency points, the acoustic reflectivity is 1, which indicates that the acoustic signal at the tunneling frequency is rarely reflected back, almost all through the whole metamaterial model; the acoustic signal outside the tunneling frequency range is almost fully reflected. Figure 11 compares the acoustic performance between the ${Th}_{z}$ = 15 cm at N=6 case and the ${Th}_{z}$ = 0 at N=3 case, and both cases agree with each other at the conditions: ${Th}_{z}$ = n×1.5 cm and N=m×3, where *n* and *m* are integers.

When the thickness of air cushion B is more than 10 times greater than PSPC air space $A_{1}$, the sub-PSPC parts can be treated as infinitely apart, and their corresponding tunneling frequency should be calculated with theoretical models in Section 3.1.

## 4. Experiment and Results

### 4.1. Experiment

**Figure 12:**
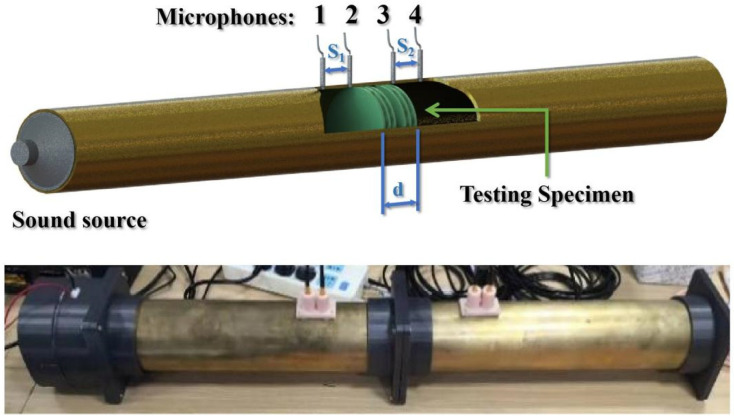
Experimental setup for ASTM E2611-17 standard impedance tube: (**top**), 3D sectional schematic; (**bottom**), corresponding physical test apparatus.

An ASTM E2611-17 standard acoustics impedance tube with an echoic ending was implied for the specimen testing, as illustrated in Figure 12 [48]. To ensure accurate acoustic characteristic measurement, a consistent performance across all equipment is key to the experimental setup. Considering the combined dynamic response of the speakers and microphones in the ASTM E2611-17 impedance tube test setup, the dynamic deviation remained within −2 dB between 50 and 800 Hz, indicating an extremely flat response. Beyond this frequency range, the perturbations of the whole system started to vary significantly, compromising measurement accuracy and precision.

A uniform sound was swiped from 50 to 800 Hz by the speaker and was projected to the testing specimen in the coaxial direction. Four 1/8″ AWA14615 measurement microphones were flushed mounted on the inner surface of the tube, and the microphone diameter is less than 20% of the measured minimum wavelength. The maximum microphone spacing is less than 80% of the required minimum half-wavelength. Four microphone cables, a Victor VC2040H signal generator, and a SAST D5-200A power amplifier were connected to the ICON Upod Nano audio signal acquisition system, enabling real-time data collection.

**Figure 13:**
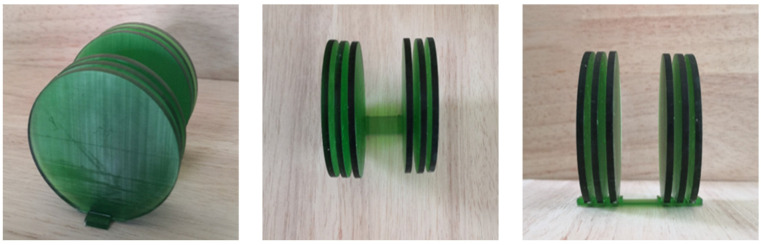
Three-dimensional-printed phononic crystal specimen (${Th}_{z}$ = 15 cm and N=6) with positioning spacer; the thicknesses of the air layer is 0.002 m, and the thicknesses of solid material is 0.005 m.

Given the predicted transmission, which is shown in Figure 7 and Figure 8a, the ${Th}_{z}$ = 2 cm at N=6 and ${Th}_{z}$ = 0.5 cm at N=6 cases were chosen as the test scenarios to match the dynamic range from 50 to 800 Hz in lieu of up to 1200 Hz. The specimen (Figure 13) was fabricated in the same size as the tube’s inner dimension and tightly sealed to the tube’s wall to minimize the transmission leakage. As shown in Figure 13, the layered phonon crystal is made by 3D printing, with three layers of 0.005 m coupled with two layers of 0.002 m. The layered structure is photosensitive resin, the density is $\rho_{2}=1300\;\mathrm{kg}/m^{3}$, the sound velocity is $c_{2}=2388\;m/s$, the middle air-layer thickness is 0.02 m, the air density is $\rho_{1}=1.205\;kg/m^{3}$, and the sound velocity is $c_{1}=343\;m/s$. The acoustic impedance of air is $Z_{A_{1}}=413.315\;Kg/\left(m^{2}\cdot s\right)$, and the photosensitive resin is $Z_{A_{2}}=310.44\times 10^{4}\;Kg/\left(m^{2}\cdot s\right)$, indicating that the resin material approximately meets the rigid body condition in the test, which is consistent with the simulation setting.

The plane wave was measured both upstream and downstream of the specimen at four locations for amplitudes and phases in order to obtain the corresponding acoustic transport matrix. Subsequently, the auto-spectrum and cross-spectrum from the transport matrix were used to calculate the transfer functions, $H_{n,ref}$, in a complex form:

(18) $$H_{n,ref}=G_{n,ref}/G_{ref,ref}$$

where $G_{ref,ref}$ is the measured sound auto-spectrum with self-reference at Microphone 1; $G_{n,ref}$ is measured sound cross-spectrum by microphone n referring to Microphone 1. Decompose the measured sound inside the tube into forward and backward traveling waves on both sides of the specimen, and the corresponding acoustic pressure (p) and particle velocity (a) on both streams of the specimen can be obtained:

(19) $$A=j\cdot (\{H_{1,ref}exp[-jk{(l}_{1})]-H_{2,ref}exp[-jk{(l}_{1}+s_{1})]\})/\left(2\sin ks_{1}\right)$$

(20) $$B=j\cdot (\{H_{2,ref}exp[+jk{(l}_{1}+s_{1})]-H_{1,ref}exp[+jk{(l}_{1})]\})/\left(2\sin ks_{1}\right)$$

(21) $$C=j\cdot (\{H_{3,ref}exp[+jk{(l}_{2}+s_{2})]-H_{4,ref}exp[+jk{(l}_{2})]\})/\left(2\sin ks_{2}\right)$$

(22) $$D=j\cdot (\{H_{4,ref}exp[-jk{(l}_{2})]-H_{3,ref}exp[-jk{(l}_{2}+s_{2})]\})/\left(2\sin ks_{2}\right)$$

(23) $$p_{up}=A+B,\;p_{dn}=Ce^{-jkd}+De^{+jkd},\;a_{up}=(A-B)/(\rho v),\;\mathrm{and}\;a_{dn}=(Ce^{-jkd}-De^{+jkd})/(\rho v)$$

The subsequent measured transfer matrix, $T_{m}$, can be obtained as follows:

(24) $$T_{m}=\left[\begin{array}{cc} T_{m11} & T_{m12}\\ T_{m21} & T_{m22} \end{array}\right]=\frac{\left[\begin{array}{cc} p_{dn}a_{dn}+p_{up}a_{up} & {p_{up}}^{2}-{p_{dn}}^{2}\\ {a_{up}}^{2}-{a_{dn}}^{2} & p_{dn}a_{dn}+p_{up}a_{up} \end{array}\right]}{p_{up}a_{dn}+p_{dn}a_{up}}$$

Then, the measured transmission rate can be calculated by the transfer matrix:

(25) $$T_{r}^{\prime \prime }=\left(2e^{jkd}\right)/\left\{T_{m11}+[T_{m12}/\left(\rho v\right)]+\rho vT_{m21}+T_{m22}\right\}$$

### 4.2. Measurement and Calculation Results

**Figure 14:**
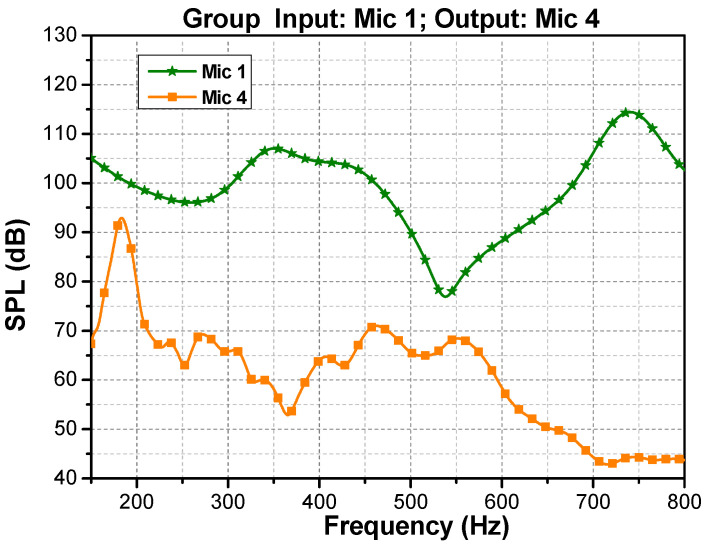
Experimentally measured input (Microphone 1) and output (Microphone 4) data.

The sound transmission of the PSPC specimen, with up- and downstream periods of N=6 and ${Th}_{z}$ = 2 cm, was experimentally measured by the impedance tube introduced in Section 4.1. Figure 14 illustrates the upstream and downstream sound-pressure level measured by Microphones 1 and 4, under 800 Hz, labeled as the input and output data.

**Figure 15:**
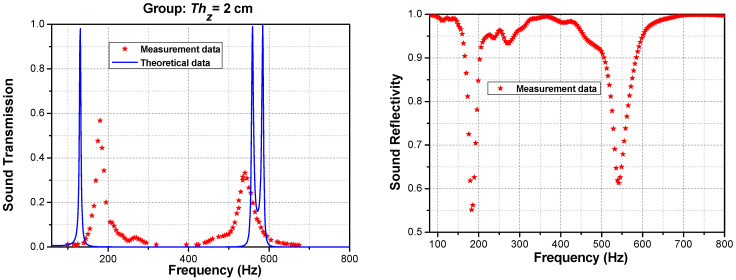
Theoretical and experimental sound transmissions at ${Th}_{z}$ = 2 cm, *N =* 6.

It can be observed that the differences between the input and output sound-pressure levels (SPLs) near 100 Hz to 200 Hz and 500 Hz to 600 Hz at the tunneling peaks do not exceed 10 dB, thus indicating high transmission. Calculation using Equations (19)–(25) shows that the measured transmission of the phononic crystal was primarily below 0.05 within 800 Hz, with exceptional tunneling transmissions of 0.56 at 189 Hz and 0.32 at 538 Hz. This result is in close agreement with the theoretical prediction from Equation (17), as clearly shown in Figure 15. As discussed previously, these two tunneling peaks distinctly demarcate a stopband from 189 Hz to 538 Hz that can be accurately predicted using the accessible geometric parameters ${Th}_{z}$ and N and the acoustic impedance properties of a single layer of hull material.

**Figure 16:**
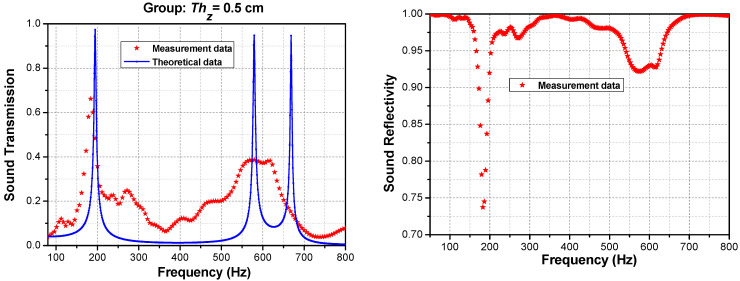
Theoretical and experimental sound transmissions and sound reflectivity at ${Th}_{z}$ = 0.5 cm, *N =* 6.

Similarly, Figure 16 shows the acoustic transmission and reflectivity for ${Th}_{z}$ = 0.5 cm. The results can be predicted for the tunneling peak frequencies of interest. The frequency deviations may arise from a random error, which can be reduced by averaging multiple measurements. The bias error may stem from a distance error between the sample and microphone, the difference between the acoustic center and geometric center of the microphone during testing, or pipe attenuation due to viscous and thermal losses as the incident and reflected sound waves propagate within the pipe. In addition, the surface texture plays an essential role in determining the acoustic properties of any material or object. For 3D-printed parts, the additive manufacturing process typically produces rougher, more uneven surfaces due to the accumulation of small errors and irregularities between layers over a vertical distance. This surface defect, in turn, impacts the acoustic properties from the experiment [49,50]. However, this analysis remains valid for studying the characteristics of tunneling frequencies in broadband stopbands. This provides a novel and convenient method for engineers to consider soundproofing characteristics during the hull design stages for ocean vehicles. Sun et al. also demonstrated that the transport loss of the mixed structure was better than using a single component [51].

Therefore, according to the theoretical prediction, FEA simulation, and experimental results, we see that, for the given two groups of specific PSPC and an arbitrary air cushion thickness, it is possible to predict the stopband and tunneling frequencies of the PSPC-layered metamaterial hull. Assuming that there are two groups of PSPC with the period of 2N sandwiching an air cushion layer and that the air cushion thickness (${Th}_{z}$) is less than 10 times of the PSPC space $A_{1}$, it should have the highest tunneling frequency, $f_{max}$, and a lower tunneling frequency, $f_{min}$, that meets $0<f_{min}<f_{max}$. It is possible to determine that there will be $2^{n}N-1$ tunneling frequencies for a $2^{n}N$ period PSPC, and these tunneling frequencies can be easily predicted by simply seeking the intersection nodes between sub-matrix terms $T_{2}$ and $T_{3}$ of Equation (16).

## 5. Conclusions

Acoustic metamaterials impact the hull of ocean vehicles by enabling stealth, sensing, and navigation capabilities through the advanced manipulation and control of noises from their powertrain. Especially in the low frequency range, insulating large band of sound and simultaneously leaving specific coordination windows are imperative to the survivability for marine and naval applications. This paper proposes a method for effectively reducing low-frequency broadband noise by using Periodic Strato-Shaped Phononic Crystals (PSPC) meta-structures. The characteristics of the phononic crystals significantly influence the efficiency of acoustic noise insulation for ocean vehicles and other applications. The distinct tunneling frequency thresholds can be predicted by adjusting the material properties. The simplicity of this meta-structure facilitates noise insulation and sound filtering for marine engineering equipment. Ultra-low noise transmission from 20 to 800 Hz greatly aids efficient noise control, using straightforward layered hull plates.

Based on theoretical and finite element analysis predictions, this paper experimentally validated the acoustic transmission through PSPC specimens with specific hull material properties, layer spacing, period number, and air cushion thickness. The tunneling frequency characteristics of phononic crystals were studied: (1) For given material properties, spacing, and number of periods, the tunneling frequencies can be efficiently predicted by determining the intersection nodes of the submatrices in the transfer matrix. (2) Based on (1), it is possible to determine that there will be $2^{n}N-1$ tunneling frequencies for a phononic crystal of $2^{n}N$ periods. (3) Conversely, the hull structural properties can be deduced from knowing the acoustic impedance of a single plate and the tunneling frequencies of the complete PSPC meta-structural hull. 

## Data Availability

Data available on request due to restrictions e.g., privacy or ethical.
